# Calorimetric analysis of ice onset temperature during cryoablation: a model approach to identify early predictors of effective applications

**DOI:** 10.1038/s41598-021-95204-2

**Published:** 2021-08-04

**Authors:** Elena Campagnoli, Andrea Ballatore, Valter Giaretto, Matteo Anselmino

**Affiliations:** 1 Department of Energy, Politecnico di Torino, Turin, Italy; 2 Division of Cardiology, “Città della Salute e della Scienza di Torino” Hospital, Department of Medical Sciences, University of Turin, corso Dogliotti 14, 10126 Turin, Italy

**Keywords:** Interventional cardiology, Computational methods

## Abstract

Aim of the present study is to analyze thermal events occurring during cryoablation. Different bovine liver samples underwent freezing cycles at different cooling rate (from 0.0075 to 25 K/min). Ice onset temperature and specific latent heat capacity of the ice formation process were measured according to differential scanning calorimetry signals. A computational model of the thermal events occurring during cryoablation was compiled using Neumann’s analytical solution. Latent heat (#1 = 139.8 ± 7.4 J/g, #2 = 147.8 ± 7.9 J/g, #3 = 159.0 ± 4.1 J/g) of all liver samples was independent of the ice onset temperature, but linearly dependent on the water content. Ice onset temperature was proportional to the logarithm of the cooling rate in the range 5 ÷ 25 K/min (#3a = − 12.2 °C, #3b = − 16.2 °C, #3c = − 6.6 °C at 5K/min; #3a = − 16.5 °C, #3b = − 19.3 °C, #3c = − 11.6 °C at 25 K/min). Ice onset temperature was associated with both the way in which the heat involved into the phase transition was delivered and with the thermal gradient inside the tissue. Ice onset temperature should be evaluated in the early phase of the ablation to tailor cryoenergy delivery. In order to obtain low ice trigger temperatures and consequent low ablation temperatures a high cooling rate is necessary.

## Introduction

The complex interactions between factors inducing and maintaining atrial fibrillation (AF) is an evolving challenge for clinicians. Catheter ablation is currently indicated to improve symptoms in patients in whom antiarrhythmic drugs (AAD) have failed or are not tolerated^1^^.^ However, new evidences advocate for an extension of its use even as first-line therapy since it appears to reduce long-term recurrences, compared to AADs^2,3^.

To date the cornerstone of catheter ablation is complete electrical isolation of the pulmonary veins by cell necrosis^4^. Aside high focused ultrasound^5^ or laser^6^, cell death is typically triggered by thermal input through the endocardial surface. In clinical practice, the most used technologies are radiofrequency (0.5 ÷ 1 MHz), through which the local temperature of the tissue is increased to about 40 °C, and cryoablation, where the involved tissue is typically cooled down below − 40 °C^7^. In both contexts, a suitable probe in direct contact with the endocardium obtains the thermal effects, and the thermal propagation inside the tissue occurs mainly by heat conduction.

Extensive literature^8–15^, devoted to compare and discuss ablation strategies, has shown that cryoablation and radiofrequency have similar efficacy and safety profiles. Since long-term effects remain the goal to be pursued, in the last decade all development steps of both approaches have been oriented to improve the procedure effectiveness in reducing recurrences. Therefore, keeping strongly in mind safety of the patient, to increase the overall success of whatever ablative techniques and, perhaps, reduce the duration of the procedure, technical solutions have been oriented to improve the probe – tissue interface energy delivery.

With this perspective, in the last years, our group has assessed cryogenic applications, investigating in-vitro ice onset and its growth in pure water, in water agar-gel^16^, and in ex vivo bovine liver^17–19^. These studies highlighted how the beginning of ice formation occurs at different supercooled states depending on contact surface, apparently related to cooling rate. Ice penetration inside the investigated materials was found linear with the square root of time. Moreover, the rate of growth of ice was found proportional to the heat flux subtracted at the probe interface, in agreement with the theoretical approach suggested by Neumann^20^ (see Appendix). Based on these experiments, it appeared that freezing above a certain cutoff of time may not be useful to improve procedure effectiveness, and might, instead, become detrimental for the surrounding tissues. In addition, similarly to what emerged from cryoablation of other arrhythmias than AF^21^, specific cryodynamic parameters (e.g. minimum temperature reached during the ablation^22^ or time to PV isolation^23,24^) may relate to acute and long-term success. The experimental outcomes were obtained in a wide temperature range, by means of a calorimetric apparatus commonly employed for the determination of latent heat and specific heat capacity of several materials. The change in these thermal properties versus temperature was the focus of the analysis, in particular during the release of heat by phase transition.

## Materials and methods

Fresh bovine livers from three slaughter animals for food use were employed for the calorimetric investigations. The procurement took place at three different stages, taking each time a massive portion of liver (100–200 g), placed to limit dehydration in a sealed container and stored at a constant temperature close to 4 °C for no more than 36 h.

Avoiding the surrounding connective tissue and the visible blood vessels, homogeneous—looking samples were picked from the inner portions of each liver. For determination of water content, samples with a mass ranging from a few tens of milligrams to a few grams were taken, while for the calorimetric measurements, samples compatible with the dimensions of the crucible and with a mass between 15 and 40 mg were used. Mass measurements were performed with an analytical Sartorius balance, model Secura^®^ 125, self-calibrating, with repeatability of ± 0.01 mg.

The water content of the liver samples was determined, as reported in^19^, by keeping the sample at a temperature around 80 °C and assuming that what evaporates is only water, so that the mass fraction of water and that of the dehydrated liver, both referred to the initial mass, are complementary. The mass fraction of water was found to be independent of the initial size of the samples^19^, so a single value was used for the samples from the same liver. To these values, which are reported in Table S1 (Appendix), an uncertainty of ± 1% was attributed, estimated on the basis of evaluations on the shape and mass of the samples, with the purpose of taking into account any additional fluid dragged into the measuring crucible from the surface of the sample.

A differential scanning calorimeter (DSC) NETZSCH DSC 214 (− 70 °C ÷ 600 °C) was used to measure both the specific heat and latent heat (Netzsch Proteus Thermal Analysis 8.0.2; software version: 10/07/2020; www.netzsch-thermal-analysis.com). This experimental apparatus is equipped with two crucibles, one which is left empty and the other which alternatively houses the sample or the reference material (sapphire 99.99% purity in our case).

The analysis was performed at the same stationary temperatures, chosen in the range + 40 °C ÷ − 70 °C, shifting from one to the other with a constant cooling/heating rate. By this methodology, the differential mode on which the calorimeter is based is, theoretically, insensitive both to external perturbations and to the dynamics of the entire system.

**Figure 1:**
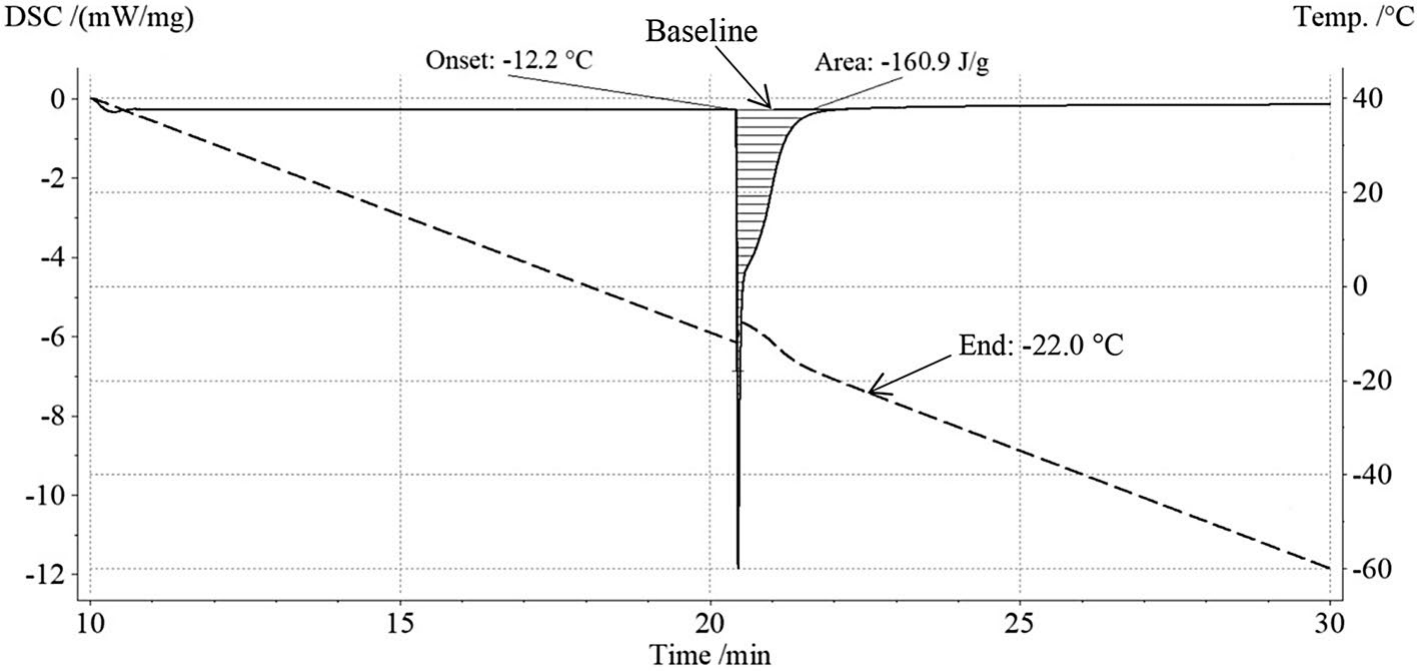
DSC signal and sample temperature vs time.

Figure 1 shows, for the liver sample named below #3a, a typical output of the calorimetric apparatus obtained in the range + 40 °C ÷ − 60 °C with an imposed cooling rate of 5 K/min. The diagram displays the DSC signal (solid line) and the actual temperature of the sample (dashed line) versus time. The DSC signal (i.e. the difference between the heat rates subtracted per unit of mass from the crucible containing the sample and the empty one) is small and approximately constant both before and after the phase transition that means in these time intervals the temperature history is consistent with the imposed cooling rate of 5 K/min. On the contrary, the sudden release of heat due to the phase transition (filled area in Fig. 1), and the consequent rapid rise in temperature (about 3 K) in the sample, requires an increase in the rate at which heat is removed in order to restore the cooling rate set.

**Figure 2:**
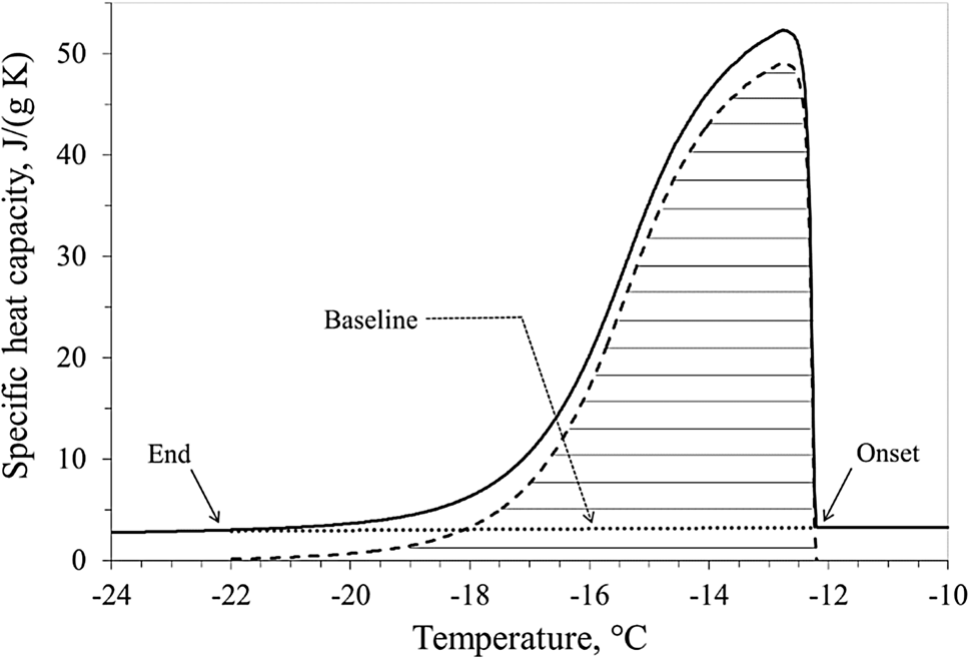
Measured and equivalent specific heat capacity vs temperature during the phase transition, obtained from DSC signal and sample temperature history shown in Fig. 1.

The solid line in Fig. 2 displays the specific heat capacity calculated by the DSC software during the phase transition shown in Fig. 1. The heat spent during this phase can be divided into two parts: the first, above the baseline introduced in Fig. 2, which is related to the phase change, and the other, below the baseline, which is associated with the decrease in sample temperature. In this way, an equivalent latent specific heat capacity is obtained which is represented in Fig. 2 by the dashed area. The areas in Figs. 1 and 2 have the same size and both are equivalent to latent heat.

## Experimental and numerical results

**Figure 3:**
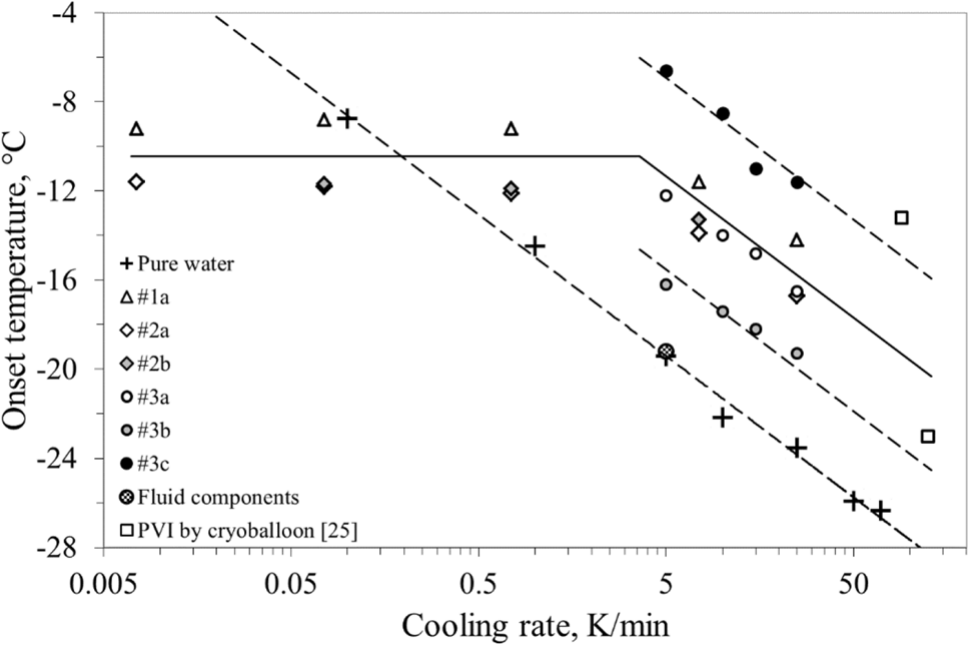
Onset temperatures vs cooling rate observed for all liver samples, including fluid components, pure water, and actual PVI by means of cryoballoon.

The beginning of the phase transition was initially investigated for pure water. Tests were performed setting different cooling rates in the range 0.1 ÷ 70 K/min, from room temperature down to − 40 °C and back to the ambient conditions. The observed onset temperatures are displayed in Fig. 3.

Six liver samples were analyzed at different cooling rates, from thermal levels close to the body temperature down to about − 70 °C. The maximum cooling rate used was 25 K/min to be sure that the calorimetric apparatus was able to reproduce exactly the desired temperature history. For the calorimetric analysis, both the fluid components (mainly serum and cellular liquid) belonging to the liver portion #3 and a dehydrated sample coming from the same liver were considered.

Table S2 (Appendix) reports the observed ice onset temperatures for the samples tested at different cooling rates. These results are also displayed in the semi-log diagram of Fig. 3, along with those for pure water and a cryoablation procedure^22^.

**Figure 4:**
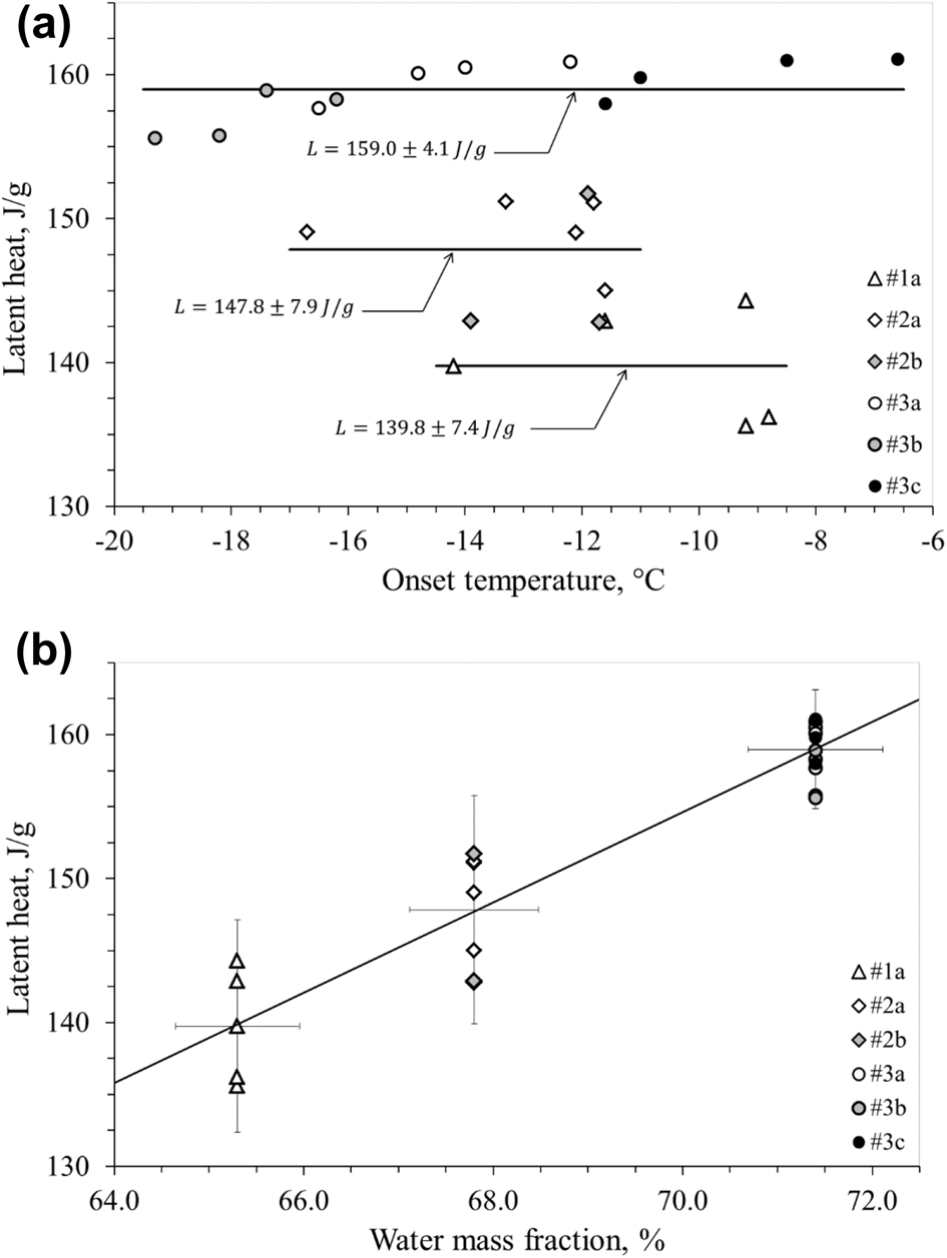
(**a**) Measured latent heat vs onset temperature of all liver samples. Solid lines represent the average latent heat values for the three liver samples. In the callouts, both the average latent heat *L* and the uncertainty at 95% confidence level are reported. (**b**) Measured latent heat vs water mass fraction of all the liver samples. Solid lines represent the linear regression. Error bars: confidence level of 95% (vertical), 1% accuracy (horizontal).

All samples, except #2b, were tested starting with the lowest cooling rate and determining at each run both the ice onset temperature and the latent heat. The latent heat measured is shown as a function of the ice onset temperature in Fig. 4a and as a function of the mass fraction of water in Fig. 4b. In Fig. 4a the solid lines represent the average latent heat for the three livers analyzed. The uncertainties in the callouts refer to the 95% confidence level of the average value displayed. In Fig. 4b, the solid lines represent the linear regression and the error bars refer to the mentioned confidence level for the latent heat and to 1% accuracy for the water mass fraction.

**Figure 5:**
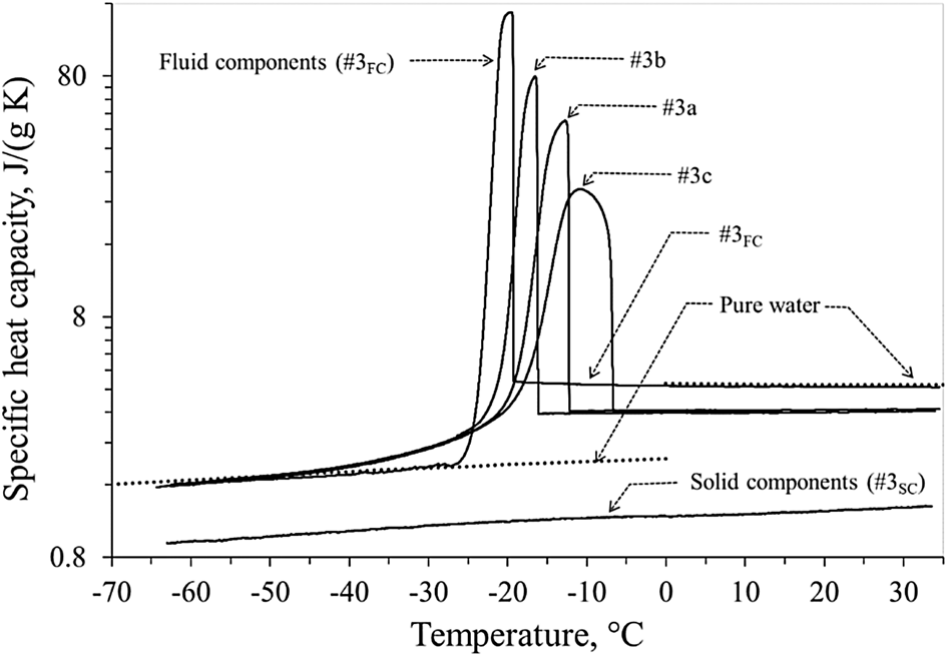
Measured specific heat capacity of liver samples #3a, #3b, and #3c, fluid (#3_FC_) and solid (#3_SC_) components, obtained with constant cooling rate of 5 K/min. Dotted lines refer to pure water values found in literature^29^.

**Figure 6:**
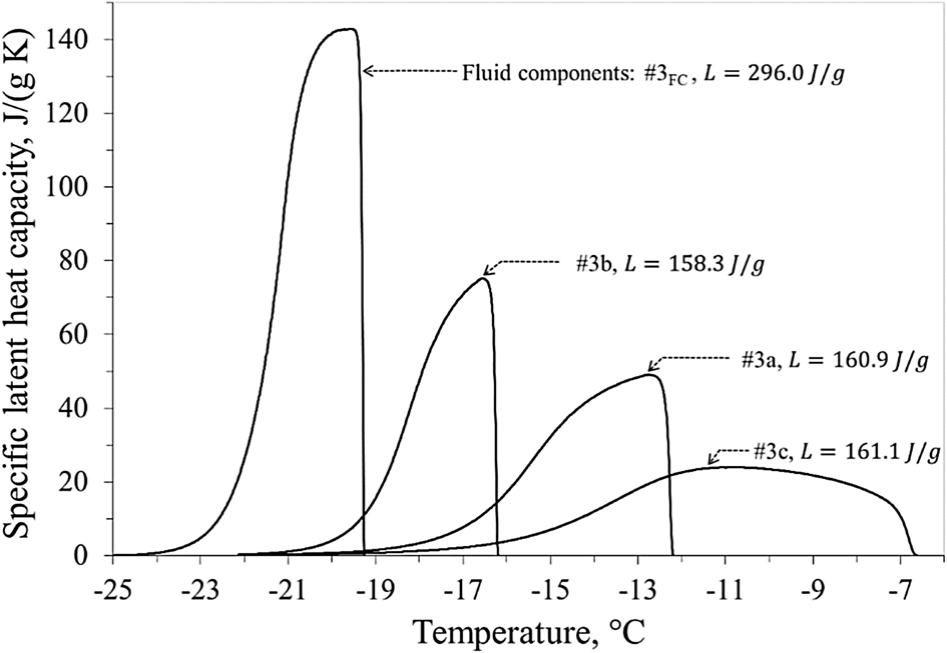
Equivalent specific latent heat capacity of liver samples #3a, #3b, #3c, and fluid components #3_FC_.

Figure 5 displays the specific heat capacity and the ice onset temperature for the samples obtained from liver #3 (samples #3a, #3b, and #3c, fluid components #3_FC_ and solid components #3_SC_) tested using a cooling rate of 5 K/min. Each of the curves shown in Fig. 5 were analyzed by introducing the relative baseline in order to evaluate both the equivalent specific latent heat capacity, shown in Fig. 6, and the end transition temperature. Quite similar end transition temperatures, close to − 22 °C, were found for liver samples #3a, #3b, and #3c, whereas in the case of fluid components, the end of the transition was around − 24.5 °C. As expected, no phase transitions was observed for solid components.

**Figure 7:**
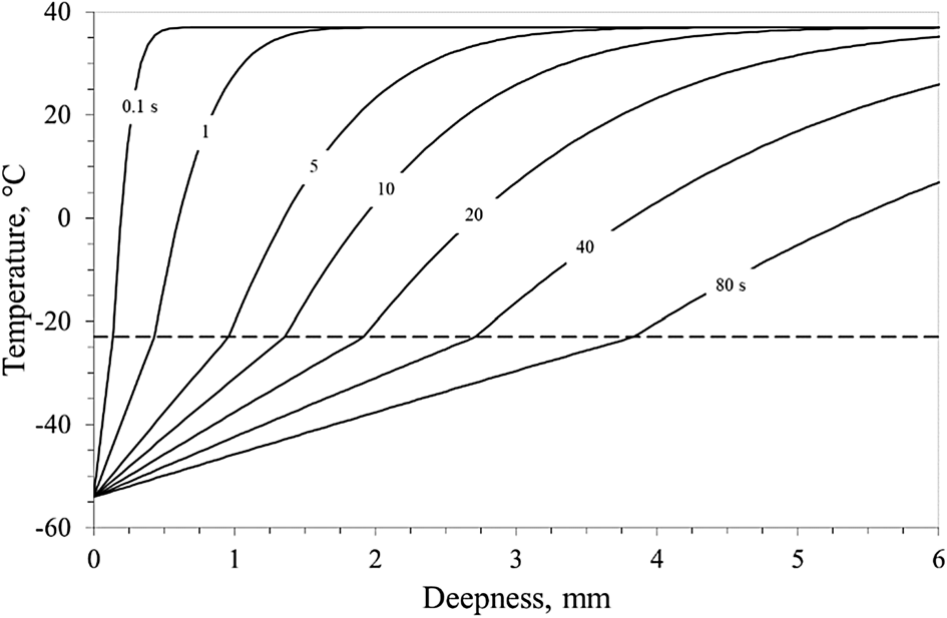
Temperature distribution versus tissue deepness at different time obtained from a constant ablative temperature of − 54 °C; dashed line refers to ice onset temperature of − 23 °C.

**Figure 8:**
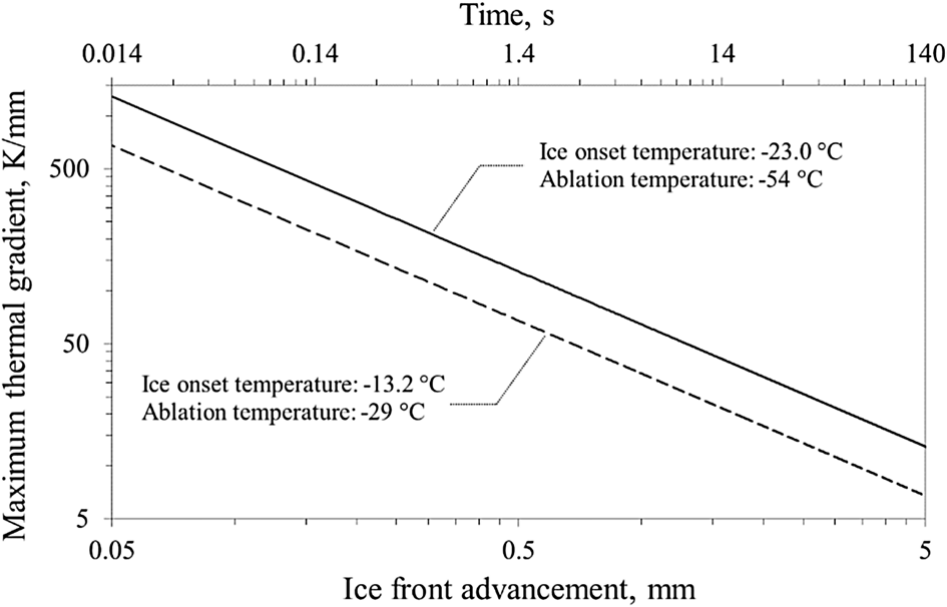
Maximum thermal gradient at the frozen–unfrozen tissue interface versus ice front advancement for different onset and ablation temperatures. The time scale regards the solid line trend and it must be about doubled for the dashed line (~ 0.028 ÷ 280 s).

Figures 7 and 8 display the numerical results obtained applying the Neumann analytical model (see Appendix) to the data reported in Figure S1.

## Discussion

An important task in cryobiology is to understand how the cooling rate and the contact between the biological tissue and the cold source affect both the heat transfer and the phase transition, to consequently predict biological outcome.

Our experimental setting initially investigated the effect of cooling rate on the ice onset temperature. For pure water the ice onset temperature was found to be clearly correlated with the cooling rate (Fig. 3) while a dissimilar behavior was recorded for the ex vivo material below and above a cooling rate of about 1 K/min. Below this value, the onset temperatures were found practically independent of the cooling rate, whereas above 5 K/min the onset temperatures were found to decrease linearly (in a semi-log diagram), with a trend similar to water. Furthermore, at the same imposed cooling rate the samples (e.g. #1a and #2a) showed different ice onset temperatures. The comparison between the results obtained for sample #2a and #2b, tested starting from the highest cooling rate, did not show substantial differences and suggests the temperature shift between samples # 1a and # 2a is likely attributable to different water content (in fact, higher for sample #2a). Conversely, samples #3b and #3c, produced with a reduced mass and smaller contact area, influenced, as expected, the ice onset temperature. At the same cooling rate, the ice onset temperature differed for these two samples by about 9 K, a similar gap to that registered between effective or ineffective clinical cryoablations (Fig. 3). This observation further demonstrates the importance of the contact between the cryoprobe and the tissue in determining the ice trigger. The actual physical features of the interface between probe and tissue during an ablative process are, however, not predictable a priori during a cryoablation procedure, but the temperature at which the ice formation begins can easily be recorded and may provide useful details on the quality of the contact with the tissue.

The detailed description of the freezing process is the focal point of the present calorimetric analysis. The latent heat, calculated here, corresponds to the total change in enthalpy, obtained by integrating the previously defined equivalent specific latent heat capacity with respect to the temperature. The latent heat of all the liver samples was assumed to be independent of the ice onset temperature (Fig. 4a), but linearly dependent on the water content of the tissue (Fig. 4b). All the measured values of the latent heat result in the range 135.6 ÷ 161.1 J/g, in line with value of 250 J/g proposed by Kim et al.^25^ (Table I, p. 1181). In Fig. 5, the specific heat capacity of the liver samples #3a, #3b and #3c show common trends, independent of the different ice trigger temperature of each sample. Pure water (dotted line in Fig. 5) and fluid components #3_FC_ have a quite similar specific heat capacity. Therefore, taking into account the measured water content (71.3% for liver #3), the specific heat capacity of unfrozen samples #3a, #3b and #3c can be verified as a linear combination between heat capacities of the fluid components #3_FC_ and the dehydrated liver #3_SC_. Figure 6 highlights how the heat involved in the phase change was delivered in temperature by the different ice triggers. The higher the ice onset temperature, the wider the temperature range required to complete the transition inside the tissue, while the lower the ice onset temperature the faster the ice forming in the tissue. The fast ice forming suggests high temperature gradients in the target tissue, with consequent high mechanical stress to obtain permanent damage to the cellular membrane^26,27^. This observation supports the clinical indication to extend the cryoenergy application for AF treatment in case the PV-cryoballoon contact is suboptimal, as the transition phase is completed in a wider temperature range. However, it can be noticed that, whereas the ice onset temperatures greatly differ among the samples, the temperature at which the transition phase is completed is close to − 22 °C, therefore low temperatures are in any case necessary. The exact temperature ranges found in this work cannot, though, be directly transposed in clinical practice, so it is not possible to determine if a sufficiently low temperature could be achieved with a poor contact ablation.

Several considerations can be drawn from these observations. In fact, it is useful to reiterate that the initial ice trigger temperature at the probe interface also determines the ice onset temperature inside the tissue, as was found experimentally^17,18^. A higher subtracted heat flux, which we have demonstrated to be associated with a lower ice onset temperature, implies a greater thermal gradient at the frozen/unfrozen tissue interface. In other words, in this scenario lower temperatures are reached in proximity of the cooling source (i.e. the cryoballoon) whereas the surrounding tissues are less affected and present warmer temperatures.

From a clinical point of view, this means that an ablation with an optimal cryoballoon–PV contact (and, therefore, a greater subtracted heat flux) appears more efficient and allows a better control of cryoenergy delivery on the target, preserving the adjacent structures. Ice onset temperature real time recording during applications, easily implementable in current clinical practice without the need of any change to the procedure, might therefore be helpful to predict effectiveness of the procedure. On the other hand, we have previously demonstrated that the ice front progression is proportional to the square root of time^18^, therefore the duration of the ablation must be shortened accordingly in order not to damage the surrounding tissues. This is particularly evident by analyzing Figs. 7 and 8: indeed, the thermal gradient at the ice front (which is represented by the slope of the different curves in the Fig. 7) dramatically decreases as the ablation proceeds. Therefore, as the ablation proceeds in the surrounding tissues, which must be protected from an excessive and unwanted cooling during the cryoenergy delivery, these undergo a faster temperature drop.

Since most of the complications related to AF cryoablation, as the fortunately rare but potentially fatal atrioesophageal fistula, are caused by a thermal injury to structures adjacent to the atrium, these observations have strong clinical implications^28^. The present analysis indicates that during cryoenergy delivery a low heat flux subtracted, associated with a poor cryoballoon–PV contact, cannot be compensated by an increase in the duration of the ablation, as the thermodynamic characteristics and, subsequently, the effects on the tissue are profoundly different. A low ablation temperature is an important predictor of ablation success, and the − 40 °C/− 50 °C range is theoretically adequate for PV isolation. However, the interval between − 40 and − 50 °C is wide, especially in clinical practice where these values refer to the plateau of the temperature curve (and therefore the time to reduce temperature is considerable). Kinetics of temperature decrease is extremely important: time to reach ablation temperature is crucial to determine the ablation success. Identifying early predictors to improve cryoenergy delivery and potentially preserve surrounding tissues from collateral damage, therefore, appears highly relevant, especially given the lack of “contact force” details for cryoprobes similar to that adopted in the radiofrequency devices^12^. In this scenario ice onset temperature surely candidates as an interesting parameter of the thermal behavior of cryoablation, performing as an early marker of inadequate PV-cryoballoon contact and possibly predicting effectiveness of the procedure. In fact, ice onset temperature real time recording during applications may be easily implemented in clinical practice, without the need of any change to the actual protocol and system. Ice onset temperature is higher than the ablation one, and easily detectable during the early phase of the cooling process. Regarding the ability to detect the onset of ice formation, it is interesting to observe registrations from clinical use of a cryoballoon (Arctic Front, 28-mm diameter, Medtronic CryoCath LP, Pointe-Claire, Quebec, Canada) for pulmonary vein isolation. As a useful example, the temperature data were deduced in a fairly accurate way from the graph reported by Furnkranz et al.^22^ (page 822), and displayed in Figure S1 (Appendix). In the figure, two recorded temperature trends versus the relative time referred to the ice trigger time *t*_0_ are shown. Different cooling rates of about − 90 K/min and − 125 K/min are observed before ice formation, and different ablation temperatures of − 29 °C and − 54 °C are displayed in the case of unsuccessful and successful procedure, respectively. Both trends show a sudden increase in temperature that could refer to the onset of the phase transition.

Eventually, the present work, analyzing the thermal behavior of a biological tissue subjected to freezing in search of the most effective and safe cooling protocol, suggests ice onset temperature could become one of the key parameters to evaluate in the early phase of AF cryoablation in order to tailor delivery of each application. The thermal gradient induced in the tissue places an important role in the effectiveness of the ablation, suggesting it probably could be useless and possibly detrimental to extend cryoenergy application to a fixed duration without considering the cryodinamic parameters. Achieving a low ice onset temperature during the initial cooling is highly relevant to achieve permanent target tissue damage in accordance with the clinical suggestions, that, albeit in a different scenario, suggest to reach low ablation temperatures with high cooling rates^21^.

### Limitations

This work presents the following limitations. Considerations arising from present results cannot be directly transposed to clinical practice. The presented model does not take into account blood flow or metabolism, but, although the analysis is limited by the use of an ex vivo tissue, the outcomes refer to a physical behavior not altered in the frozen state by other superimposed effects (i.e. metabolic heat and blood flow), faithfully mimicking thermal effects of a cryoballoon catheter used in real practice. In addition, compared to a standard cryoablation our experiments were conducted at a lower cooling rate: in fact, a higher cooling rate would not have allowed properly measurements with the calorimetric apparatus.

Liver and heart tissue can differ in terms of water percentage, electrolyte concentration and inter-cellular connection, however the aim of this paper is to highlight predictors of adequate cryoenergy application during the early phases of delivery, and, in this respect, the biophysical properties of the tissues do not seem to influence the beginning of ice formation. Therefore, aware that the measures of the present work will perhaps necessitate correction before being transposed to the heart tissue, the general laws appear valid and, at least, pave the way to further clinical studies.

## Conclusion

Ice onset temperature, which, to the best of our knowledge, has never been evaluated as a predictor of cryoablation success, appears as a relevant indicator of the subtracted heat flux and holds the potential to predict, in the early phase of the application, the minimum temperature reached. The lower the temperature at which the ice is triggered, the stronger the thermal effect produced in the biological tissue involved.

These findings hold the potential to improve safety profile of cryoablation application and warrant further researches, both clinical and in laboratory, in order to confirm the optimal freezing kinetics, and, eventually, guide technical amendments to current cryoprobes (e.g. higher imposed cooling rate).

## Supplementary Information

Supplementary Information.

## Data Availability

The data underlying this article will be shared on reasonable request to the corresponding author.

## References

[1] Hindricks G (2020). 2020 ESC Guidelines for the diagnosis and management of atrial fibrillation developed in collaboration with the European Association of Cardio-Thoracic Surgery (EACTS). Eur. Heart J..

[2] Saglietto A, Gaita F, De Ponti R, De Ferrari GM, Anselmino M (2021). Catheter ablation vs. anti-arrhythmic drugs as first-line treatment in symptomatic paroxysmal atrial fibrillation: A systematic review and meta-analysis of randomized clinical trials. Front. Cardiovasc. Med..

[3] Kirchhof P (2020). Early rhythm-control therapy in patients with atrial fibrillation. N. Engl. J. Med..

[4] Calkins H (2017). 2017 HRS/EHRA/ECAS/APHRS/SOLAECE expert consensus statement on catheter and surgical ablation of atrial fibrillation. Heart Rhythm.

[5] Natale A (2000). First human experience with pulmonary vein isolation using a through-the-balloon circumferential ultrasound ablation system for recurrent atrial fibrillation. Circulation.

[6] Schmidt B (2010). Feasibility of circumferential pulmonary vein isolation using a novel endoscopic ablation system. Circ. Arrhythmia Electrophysiol..

[7] Hong KL, Borges J, Glover B (2020). Catheter ablation for the management of atrial fibrillation: Current technical perspectives. Open Heart.

[8] Luik A (2015). Cryoballoon versus open irrigated radiofrequency ablation in patients with paroxysmal atrial fibrillation. Circulation.

[9] Wasserlauf J (2015). Cryoballoon versus radiofrequency catheter ablation for paroxysmal atrial fibrillation. PACE Pacing Clin. Electrophysiol..

[10] Aryana A (2015). Acute and long-term outcomes of catheter ablation of atrial fibrillation using the second-generation cryoballoon versus open-irrigated radiofrequency: A multicenter experience. J. Cardiovasc. Electrophysiol..

[11] Hunter RJ (2015). Point-by-point radiofrequency ablation versus the cryoballoon or a novel combined approach: A randomized trial comparing 3 methods of pulmonary vein isolation for paroxysmal atrial fibrillation (the cryo versus RF trial). J. Cardiovasc. Electrophysiol..

[12] Squara F (2015). Comparison between radiofrequency with contact force-sensing and second-generation cryoballoon for paroxysmal atrial fibrillation catheter ablation: A multicentre European evaluation. Europace.

[13] Jourda F (2014). Contact-force guided radiofrequency vs. second-generation balloon cryotherapy for pulmonary vein isolation in patients with paroxysmal atrial fibrillation—A prospective evaluation. Europace.

[14] Kuck K-H (2016). Cryoballoon or radiofrequency ablation for paroxysmal atrial fibrillation. N. Engl. J. Med..

[15] Matta M, Anselmino M, Ferraris F, Scaglione M, Gaita F (2018). Cryoballoon vs. radiofrequency contact force ablation for paroxysmal atrial fibrillation: A propensity score analysis. J. Cardiovasc. Med..

[16] Giaretto V, Passerone C (2017). Mirror image technique for the thermal analysis in cryoablation: Experimental setup and validation. Cryobiology.

[17] Giaretto V, Passerone C (2018). Experimental investigation on the ice formation and growth in ex vivo bovine liver. Am. J. Biosci..

[18] Giaretto V (2019). Thermodynamic properties of atrial fibrillation cryoablation: A model-based approach to improve knowledge on energy delivery. J. R. Soc. Interface.

[19] Campagnoli E, Giaretto V (2021). Experimental investigation on thermal conductivity and thermal diffusivity of ex-vivo bovine liver from room temperature down to −60 °C. Materials..

[20] Carslaw H, Jaeger J (1959). Conduction of Heat in Solids.

[21] Matta M (2017). Cooling dynamics: A new predictor of long-term efficacy of atrioventricular nodal reentrant tachycardia cryoablation. J. Interv. Cardiol. Electrophysiol..

[22] Fürnkranz A (2011). Cryoballoon temperature predicts acute pulmonary vein isolation. Heart Rhythm.

[23] Ciconte G (2015). On the quest for the best freeze. Circ. Arrhythmia Electrophysiol..

[24] Aryana A (2016). Procedural and biophysical indicators of durable pulmonary vein isolation during cryoballoon ablation of atrial fibrillation. Heart Rhythm.

[25] Kim C, O’Rourke AP, Will JA, Mahvi DM, Webster JG (2008). Finite-element analysis of hepatic cryoablation around a large blood vessel. IEEE Trans. Biomed. Eng..

[26] Poornejad N (2015). Freezing/thawing without cryoprotectant damages native but not decellularized porcine renal tissue. Organogenesis.

[27] Steif PS, Palastro MC, Rabin Y (2007). The effect of temperature gradients on stress development during cryopreservation via vitrification. Cell Preserv. Technol..

[28] Sarairah SY (2020). Esophageal thermal injury following cryoballoon ablation for atrial fibrillation. JACC Clin. Electrophysiol..

[29] Fukusako S, Yamada M (1993). Recent advances in research on water-freezing and ice-melting problems. Exp. Therm. Fluid Sci..

