# Supplementary material for: Calorimetric analysis of ice onset temperature during cryoablation: a model approach to identify early predictors of effective applications
Source: Sci Rep. 2021 Aug 4;11:15798. doi: 10.1038/s41598-021-95204-2 (PMC8339075; doi:10.1038/s41598-021-95204-2)
Supplement: Supplementary file 1 — Supplementary Information. [file 41598_2021_95204_MOESM1_ESM.docx]

**Calorimetric analysis of ice onset temperature during cryoablation: a model approach to identify early predictors of effective applications**

*Supplementary Material*

**Appendix**

**Supplementary Methods and Results**

As seen in the main discussion, the latent heat is quickly released inside tissue when the ice onset temperature is low enough, and this scenario is most likely achieved with high cooling rates. Supposing that the latter is so high that the ablation temperature is reached instantaneously, the analytical solution proposed by Neumann, summarized below can be employed for evaluating the thermal effects inside the tissue.

This limit condition was explored by assigning a uniform initial metabolic temperature $T_{body}$ throughout the depth of the tissue and a constant ablation temperature $T_{ab}$ at the cryoprobe interface. The ice onset temperature $T_{on}$ was supposed greater than the ablation one $T_{ab}$. Since the latter is reached instantaneously, consequently at the probe – tissue interface the ice is formed promptly at time $t=0$, while inside the tissue the ice formation begins when the temperature $T_{on}$ is reached.

In an attempt to make the theoretical results more realistic, a metabolic temperature $T_{body}=37^{\circ}C$ was assigned, and an ablation temperature $T_{ab}=-54^{\circ}C$ and an ice onset temperature $T_{on}=-23^{\circ}C$ were set in agreement with the effective procedure mentioned in ^1^⁠. The calorimetric and the thermophysical properties for frozen and unfrozen tissue are less important than the previous mentioned temperatures. So, taking into account the assumptions inherent to the model, the values of these properties were considered temperature independent. The values found here were assigned to the latent heat and the specific heat capacity, whereas the typical values noticed in the literature and mentioned below were considered for the thermal conductivity and thermal diffusivity.

Figure 7 shows the temperature distributions inside the tissue, obtained by using Eq. (A.5) and Eq. (A.6) at various time. Each of these trends starts from the assigned ablation temperature $T_{ab}$ and tends to the asymptotic value $T_{body}$. The dashed line in the figure represents the ice onset temperature $T_{on}$; below this value, the tissue is frozen, while it is unfrozen above. At a given time value, the temperature gradient (the slope in this diagram) is quite constant in the frozen state and it decreases by increasing the time. The maximum temperature gradient occurs at the interface between frozen – unfrozen tissue, and pertains to the unfrozen tissue. It is evident in the plot that the lower the ice onset temperature $T_{on}$, the higher the gradient in the incipient ice formation on that interface.

By using Eq. (A.10), the maximum thermal gradient inside the tissue versus ice front advancement is drawn in Figure 8 for different onset and ablation temperatures. Solid line refers to the theoretical results shown in Figure 7, whereas the dashed line was obtained using the temperatures values ($T_{on}=-13.2^{\circ}C,T_{ab}=-29^{\circ}C$) which refer to a procedure mentioned as ineffective ^1^.

Both the trends in the log – log plot are linear with a slope of -1 and consequently with a gradient decay versus ice penetration that is hyperbolic. In fact, for an ice front penetration of about half a millimeter, for both the displayed cases the temperature gradient decays tenfold. Since, the time scale showed in the upper part of the diagram refers to the solid line trend only, and the reference time for the dashed line must be about doubled, the reduction in the aforementioned gradient occurs after about one second for $T_{ab}=-54^{\circ}C$, and after a couple of seconds for $T_{ab}=-29^{\circ}C$.

Neumann’s solution used for the change of state in a semi-infinite slab of a continuous material ($x>0$) is detailed in ^2^. $T$ is the temperature at a generic position $x$ and time $t$; $T_{body}$ is the body temperature, $T_{on}$ and $T_{ab}$ ($T_{on}>T_{ab}$) are the previously mentioned ice onset and ablation temperatures, respectively.

The solution is given for the reduced temperature $\vartheta=T-T_{ab}$, and the reduced ice onset temperature is $\vartheta_{on}=T_{on}-T_{ab}$. The material properties are assumed temperature independent: $\lambda$ is the thermal conductivity, $a$ the thermal diffusivity, $c$ the specific heat capacity, and subscripts $u$ and $f$ refer to unfrozen and frozen state, respectively. $L$ is the latent heat, and at a given time $t$ the position of ice front inside tissue is $x_{I}$. The material slab is initially unfrozen at the constant temperature $\vartheta_{\infty}=T_{body}-T_{ab}.$ Surface at $x=0$ maintained at $\vartheta_{0}=0$ whatever the time $t>0$, and the applied boundary conditions are:

$x\to\infty\Rightarrow\vartheta_{u}\to\vartheta_{\infty}$; $x=0\Rightarrow\vartheta_{f}=T-T_{ab}\equiv\vartheta_{0}=0$. (A.1)

The solution depends on a dimensionless constant $\delta$ obtained with the following constraint equation:

$\frac{exp\left( -\delta^{2} \right)}{erf\left( \delta\right)}-\frac{\lambda_{u}\sqrt{a_{f}}\left( \vartheta_{\infty}-\vartheta_{on} \right)exp\left( \frac{-a_{f}}{a_{u}}\delta^{2} \right)}{\lambda_{f}\sqrt{a_{u}}\vartheta_{on}erfc\left( \delta\sqrt{\frac{a_{f}}{a_{u}}} \right)}-\frac{\delta L\sqrt{\pi}}{c_{f}\vartheta_{on}}=0,$ (A.2)

where erf(·) and erfc(·) are the error function and the complementary error function, respectively.

For example, with $T_{body}=37^{\circ}C$, $T_{on}=-23^{\circ}C$, $T_{ab}=-54^{\circ}C$,$c_{f}=3000$ J/(kg K), $\lambda_{u}=0.49$ W/(m K), $\lambda_{f}=1.40$W/(m K), $a_{u}=0.49\cdot$10^-7^ m^2^/s, $a_{f}=1.46\cdot$10^-7^ m^2^/s, and $L=160000$J/kg, by using a bisection procedure, the solution of Eq. (A.2) gives $\delta\cong0.256$.

The solution for the ice front advancement is

$x_{I}=2\delta\sqrt{a_{f}t}$, (A.3)

and the solutions for the reduced temperatures are

$\vartheta=\frac{\vartheta_{on}}{erf\left( \delta\right)}erf\left( \frac{x}{2\sqrt{a_{f}t}} \right),\forall x\leq x_{I}$, (A.5)

$\vartheta=\vartheta_{\infty}-\frac{\vartheta_{\infty}-\vartheta_{on}}{erfc\left( \delta\sqrt{\frac{a_{f}}{a_{u}}} \right)}erfc\left( \frac{x}{2\sqrt{a_{u}t}} \right),\forall x\geq x_{I}$. (A.6)

From Eq. (A.5) and Eq. (A.6) the thermal gradients at a given $x$ position result

$\frac{d\vartheta}{dx}=\frac{\vartheta_{on}}{erf\left( \delta\right)}\frac{1}{\sqrt{{\pi a}_{f}t}}exp\left( \frac{-x^{2}}{{4a}_{f}t} \right),\forall x\leq x_{I}$, (A.7)

$\frac{d\vartheta}{dx}=\frac{\vartheta_{\infty}-\vartheta_{on}}{erfc\left( \delta\sqrt{\frac{a_{f}}{a_{u}}} \right)}\frac{1}{\sqrt{{\pi a}_{u}t}}exp\left( \frac{-x^{2}}{{4a}_{u}t} \right),\forall x\geq x_{I}$, (A.8)

and at the ice front position $x_{I}$ become

$\left. \frac{d\vartheta}{dx} \right|_{x=x_{I}}=\frac{\vartheta_{on}}{erf\left( \delta\right)}\frac{1}{\sqrt{{\pi a}_{f}t}}exp\left( -\delta^{2} \right),$ for the frozen side, (A.9)

$\left. \frac{d\vartheta}{dx} \right|_{x=x_{I}}=\frac{\vartheta_{\infty}-\vartheta_{on}}{erfc\left( \delta\sqrt{\frac{a_{f}}{a_{u}}} \right)}\frac{1}{\sqrt{{\pi a}_{u}t}}exp\left( \frac{-a_{f}}{a_{u}}\delta^{2} \right),$ for the unfrozen side. (A.10)

**Supplementary Tables and Figures**

**Table S1.** Measured water mass fraction

| Bovine liver | Reference water mass fraction, $x_{W}$ |
| --- | --- |
| #1 | 0.653 ± 0.007 |
| #2 | 0.678 ± 0.007 |
| #3 | 0.713 ± 0.007 |

**Table S2.** Liver samples investigated at different cooling rates

| Liver | Sample | Mass (mg) | Cooling rate (K/min) and corresponding ice onset temperature | | | | | | | |  |
| --- | --- | --- | --- | --- | --- | --- | --- | --- | --- | --- | --- |
|  |  |  | 0.0075 | 0.075 | 0.75 | 5 | 7.5 | 10 | 15 | 25 |  |
| #1 | #1a | 40.05 | -9.2 °C | -8.8 °C | -9.2 °C | ⎯ | -11.6 °C | ⎯ | ⎯ | -14.2 °C |  |
| #2 | #2a | 36.75 | -11.6 °C | -11.8 °C | -12.1 °C | ⎯ | -13.3 °C | ⎯ | ⎯ | -16.7 °C |  |
|  | #2b | 39.86 | ⎯ | -11.8 °C | -11.9 °C | ⎯ | -13.9 °C | ⎯ | ⎯ | ⎯ |  |
| #3 | #3a | 31.91 | ⎯ | ⎯ | ⎯ | -12.2 °C | ⎯ | -14.0 °C | -14.8 °C | -16.5 °C |  |
|  | #3b | 16.80 | ⎯ | ⎯ | ⎯ | -16.2 °C | ⎯ | -17.4 °C | -18.2 °C | -19.3 °C |  |
|  | #3c | 15.34 | ⎯ | ⎯ | ⎯ | -6.6 °C | ⎯ | -8.5 °C | -11.0 °C | -11.6 °C |  |
|  | #3_FC_^§^ | 19.53 | ⎯ | ⎯ | ⎯ | -19.2 °C | ⎯ | ⎯ | ⎯ | ⎯ |  |
| ^§^ Fluid Components only. | | | | | | | | | | | |

**Figure S1.** Temperature trends recorded by a cryoballoon (Arctic Front, 28-mm diameter, Medtronic CryoCath LP, Pointe-Claire, Quebec, Canada) in the case of successful (ablation temperature -54 °C) and unsuccessful (ablation temperature -29 °C) pulmonary vein isolation. The temperature values versus time were deduced from the graph reported by Furnkranz et al. (^1^⁠, page 822)


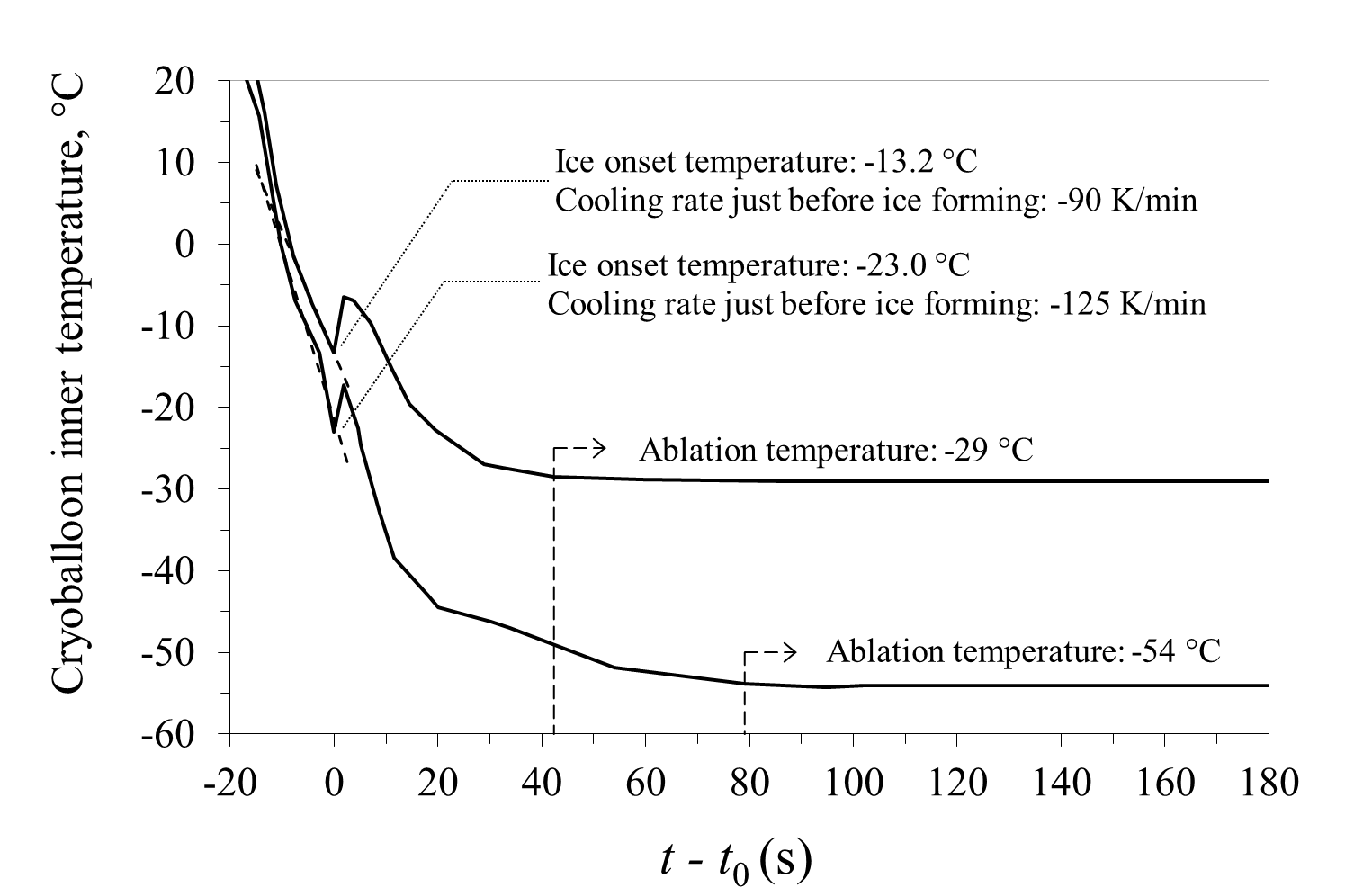


**References**

1. Fürnkranz, A. *et al.* Cryoballoon temperature predicts acute pulmonary vein isolation. *Hear. Rhythm* **8**, 821–825 (2011).

2. Carslaw, H. & Jaeger, J. *Conduction of Heat in Solids*. (Oxford University Press, 1959).
